# FlyRNAi.org—the database of the Drosophila RNAi screening center and transgenic RNAi project: 2021 update

**DOI:** 10.1093/nar/gkaa936

**Published:** 2020-10-26

**Authors:** Yanhui Hu, Aram Comjean, Jonathan Rodiger, Yifang Liu, Yue Gao, Verena Chung, Jonathan Zirin, Norbert Perrimon, Stephanie E Mohr

**Affiliations:** Department of Genetics, Blavatnik Institute, Harvard Medical School, 77 Avenue Louis Pasteur, Boston, MA 02115, USA; Drosophila RNAi Screening Center, Harvard Medical School, 77 Avenue Louis Pasteur, Boston, MA 02115, USA; Department of Genetics, Blavatnik Institute, Harvard Medical School, 77 Avenue Louis Pasteur, Boston, MA 02115, USA; Drosophila RNAi Screening Center, Harvard Medical School, 77 Avenue Louis Pasteur, Boston, MA 02115, USA; Department of Genetics, Blavatnik Institute, Harvard Medical School, 77 Avenue Louis Pasteur, Boston, MA 02115, USA; Drosophila RNAi Screening Center, Harvard Medical School, 77 Avenue Louis Pasteur, Boston, MA 02115, USA; Department of Genetics, Blavatnik Institute, Harvard Medical School, 77 Avenue Louis Pasteur, Boston, MA 02115, USA; Drosophila RNAi Screening Center, Harvard Medical School, 77 Avenue Louis Pasteur, Boston, MA 02115, USA; Department of Genetics, Blavatnik Institute, Harvard Medical School, 77 Avenue Louis Pasteur, Boston, MA 02115, USA; Drosophila RNAi Screening Center, Harvard Medical School, 77 Avenue Louis Pasteur, Boston, MA 02115, USA; Department of Genetics, Blavatnik Institute, Harvard Medical School, 77 Avenue Louis Pasteur, Boston, MA 02115, USA; Drosophila RNAi Screening Center, Harvard Medical School, 77 Avenue Louis Pasteur, Boston, MA 02115, USA; Department of Genetics, Blavatnik Institute, Harvard Medical School, 77 Avenue Louis Pasteur, Boston, MA 02115, USA; Drosophila RNAi Screening Center, Harvard Medical School, 77 Avenue Louis Pasteur, Boston, MA 02115, USA; Department of Genetics, Blavatnik Institute, Harvard Medical School, 77 Avenue Louis Pasteur, Boston, MA 02115, USA; Drosophila RNAi Screening Center, Harvard Medical School, 77 Avenue Louis Pasteur, Boston, MA 02115, USA; Howard Hughes Medical Institute, 77 Avenue Louis Pasteur, Boston, MA 02115, USA; Department of Genetics, Blavatnik Institute, Harvard Medical School, 77 Avenue Louis Pasteur, Boston, MA 02115, USA; Drosophila RNAi Screening Center, Harvard Medical School, 77 Avenue Louis Pasteur, Boston, MA 02115, USA

## Abstract

The FlyRNAi database at the Drosophila RNAi Screening Center and Transgenic RNAi Project (DRSC/TRiP) provides a suite of online resources that facilitate functional genomics studies with a special emphasis on *Drosophila melanogaster*. Currently, the database provides: gene-centric resources that facilitate ortholog mapping and mining of information about orthologs in common genetic model species; reagent-centric resources that help researchers identify RNAi and CRISPR sgRNA reagents or designs; and data-centric resources that facilitate visualization and mining of transcriptomics data, protein modification data, protein interactions, and more. Here, we discuss updated and new features that help biological and biomedical researchers efficiently identify, visualize, analyze, and integrate information and data for *Drosophila* and other species. Together, these resources facilitate multiple steps in functional genomics workflows, from building gene and reagent lists to management, analysis, and integration of data.

## INTRODUCTION

The FlyRNAi database at the Drosophila RNAi Screening Center and Transgenic RNAi Project (DRSC/TRiP) was initially developed to support genome-wide RNAi screening in cultured cells from *Drosophila melanogaster* (hereafter, *Drosophila*) (1). Now, more than fifteen years later, the database and associated online software tools and data sets have expanded in scope and utility. As a result, the FlyRNAi database has grown from a RNAi reagent and dataset tracking laboratory information management system (LIMS) to a suite of searchable user interfaces, online software tools, data sets, and more that serve a variety of purposes.

Our roots in large-scale screening have shaped our bioinformatic approaches, which include support for reagent design and management, analysis, and integration of large-scale datasets. Moreover, we have maintained our close ties to bench research, aiming to meet practical needs of biological and biomedical researchers working in *Drosophila* and other systems, and responding to input from the community with regards to improvement and new development. Community needs motivated, for example, development in 2011 of the DRSC Integrative Ortholog Prediction Tool (DIOPT) (2), our most-used resource, which has since been expanded and powers ortholog mapping in other resources, both within our suite of tools and at other sites.

The content of the FlyRNAi database has grown significantly with the addition of more algorithms and model organism data as well as many new search features added based on user feedback. At the same time, we continue to support *Drosophila* reagent design and cell-based screen data management, analysis, and integration, and transfer screen data stored in FlyRNAi to NCBI PubChem BioAssays for public access in that meta-database.

We expand on new features of our database and associated suite of tools below.

### Updates to the website

Over the years, the DRSC informatics group has implemented >30 online software tools, data resources, and so on (hereafter, ‘resources’). Each resource has a unique focus and was developed to address a different need in the scientific community. The majority of our online tools were built following a three-tier model, with a web-based user-interface at the front end, the FlyRNAi database at the backend, and business logic in a middle tier communicating between the front and back ends. The backend information is stored as many different tables of FlyRNAi database. There are tables specifically designed for each tool to accommodate the various data structures needed. At the same time, many tools also share tables that are actively maintained and updated, such as tables with gene ID mapping, transcript or protein sequences, or protein domain information. At the user interface, most tools are interconnected by genes or reagents, facilitating transitions between interfaces. For example, ortholog search results at DIOPT are linked to an RNAi reagent search page at UP-TORR, facilitating reagent identification following an ortholog identification step.

**Figure 1.**
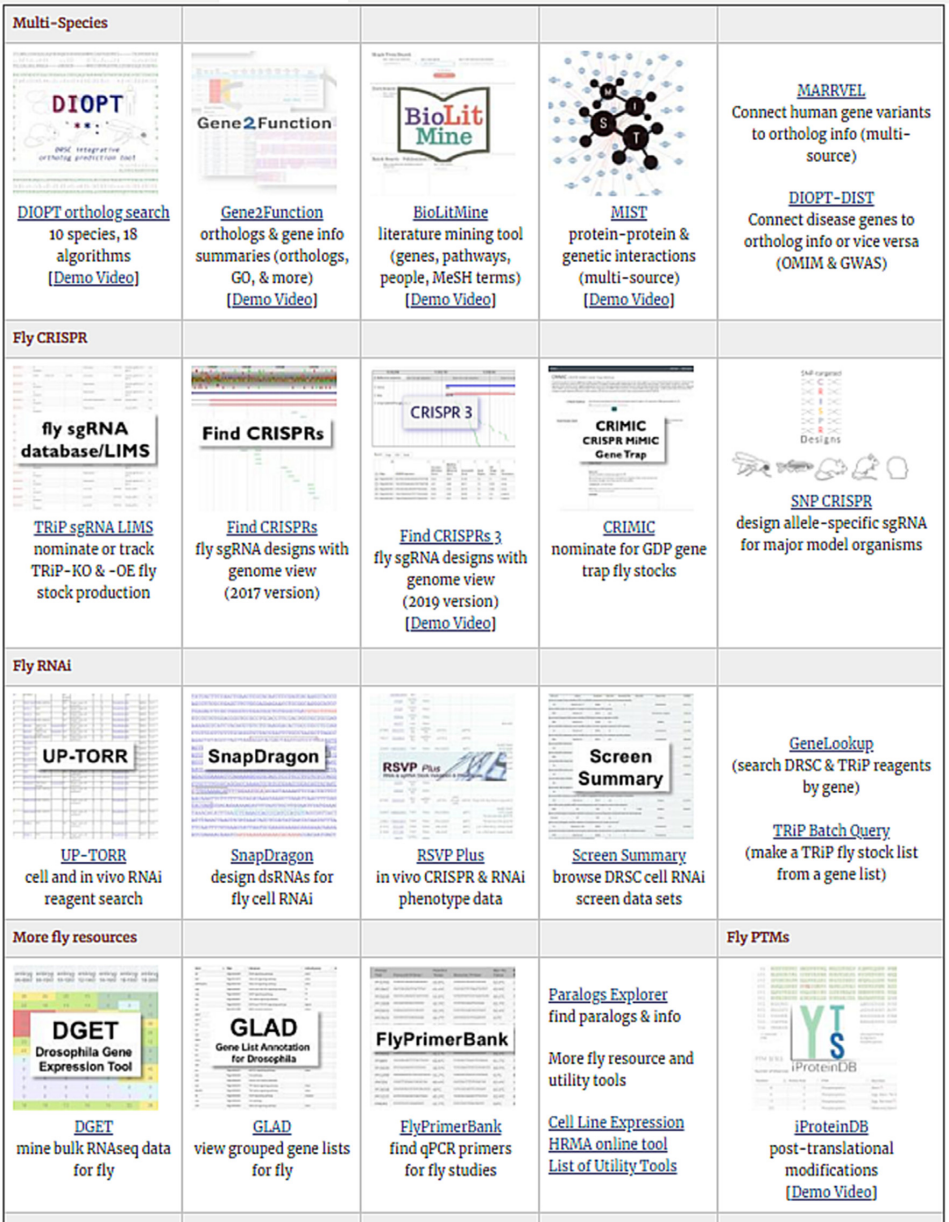
New look of the DRSC/TRiP online tools landing page.

To help users find information of interest, we have implemented several user interfaces that function independently. Since our last NAR update (3), we replaced a list of resources on our ‘overview’ page with a table that includes a graphic representation of each tool, brief explanatory text and a hyperlink to the resource (https://fgr.hms.harvard.edu/tools) (Figure 1). DRSC online resources can be grouped into three major categories: gene-centric resources, reagent-centric resources, and data-centric resources. Since the 2017 update, we have made progress in each category (Tables 1-3). The gene-centric resources help users find gene annotations and/or relationships among genes. The reagent-centric resources help users design or identify various type of reagents, as well as access information related to the quality of the reagents. The data-centric resources allow users to analyze large-scale datasets or allow for view, download, and/or mining of different types of biological data, including data from cell-based screens, in vivo phenotype data, transcriptional profiles, and proteomics data.

**Table 1.** Gene-centric resources associated with the FlyRNAi database

Resource, purpose, species supported, URL
Resource: Gene Lookup (1,37)*
Purpose: Search gene information, DRSC and TRiP reagents, and DRSC screen data
Species supported: *Drosophila*
URL: https://www.flyrnai.org/genelookup
Resource: DIOPT (2)
Purpose: Ortholog and paralog searches (single gene or batch mode)
Species supported: *Arabidopsis, C. elegans, Drosophila, human, mouse, rat, S. cerevisiae, S. pombe, X. tropicalis, zebrafish*
URL: https://www.flyrnai.org/diopt
Resource: DIOPT-DIST (2)
Purpose: Model species-to-human ortholog search with human disease associations from OMIM and MeSH (single gene or batch mode)
Species supported: *Arabidopsis, C. elegans, Drosophila, human, mouse, rat, S. cerevisiae, S. pombe, X. tropicalis, zebrafish*
URL: https://www.flyrnai.org/diopt-dist
Resource: GLAD (38)**
Purpose: Determine if a gene is a member of a list (e.g. of transcription factors or of signal transduction pathway components), enrichment analysis of a user-provided list
Species supported: *Drosophila*
URL: https://www.flyrnai.org/tools/glad/web/
Resource: Gene2Function (15)*
Purpose: Ortholog search with summary information about gene and protein function, e.g. gene ontology terms, ‘omics data, publications, etc., from MODs
Species supported: *C. elegans, Drosophila, human, mouse, rat, S. cerevisiae, S. pombe, X. tropicalis, zebrafish*
URL: http://www.gene2function.org/search/
Resource: BioLitMine (https://www.biorxiv.org/content/10.1101/2020.07.17.208249v1)**
Purpose: Literature mining with MeSH term integration (single gene search) and batch-mode search for gene-associated publications
Species supported: *Arabidopsis, C. elegans, Drosophila, human, mouse, rat, S. cerevisiae, S. pombe, X. tropicalis, zebrafish*
URL: https://www.flyrnai.org/tools/biolitmine/web/

Note:

*: can also be used for reagent identification and data mining.

**: can also be used as data-centric tool, e.g. for enrichment analysis.

**Table 2.** Reagent-centric resources associated with the FlyRNAi database

Resource, purpose, species supported, URL
Resource: Snapdragon (37)
Purpose: Design of double-stranded RNAs (dsRNAs) for cell-based RNAi knockdown
Species supported: *Drosophila* (or any sequence)
URL: https://www.flyrnai.org/cgi-bin/RNAi_find_primers.pl
Resource: FlyPrimerBank (39)
Purpose: Identification of pre-computed primers for quantitative PCR (qPCR)
Species supported: *Drosophila*
URL: https://www.flyrnai.org/cgi-bin/DRSC_primerbank.pl
Resource: Updated Targets of RNAi Reagents (UP-TORR) (40)
Purpose: Identify cell-based and *in vivo* RNAi reagents and associated information such as isoform specificity and predicted off-targets, based on updated gene annotations
Species supported: *C. elegans, Drosophila*, human, mouse
URL: https://www.flyrnai.org/up-torr/
Resource: RSVP Plus (24,41)
Purpose: Find tissue-specific validation and phenotype data for RNAi and sgRNA fly stocks
Species Supported: *Drosophila*
URL: https://www.flyrnai.org/cgi-bin/RSVP_search.pl
Resource: TRiP gRNA Fly Stock Database (24)
Purpose: LIMS and nomination portal for TRiP sgRNA fly stock production useful for CRISPR knockout or CRISPR overexpression (TRiP-KO and TRiP-OE flies)
Species Supported: *Drosophila*
URL: https://www.flyrnai.org/tools/grna_tracker/web/
Resource: CRIMIC page (25)
Purpose: Nominate genes for CRIMIC gene trap fly stock production by the *Drosophila* Gene Disruption Project (collaboration among the Bellen, Spradling and Perrimon labs)
Species supported: *Drosophila*
URL: https://www.flyrnai.org/tools/crimic/web/
Resource: Find CRISPRs (2017) (42)
Purpose: Search pre-computed sgRNAs, with genome browser view
Species Supported: *Drosophila*
URL: https://www.flyrnai.org/crispr/
Resource: Find CRISPRs 3 (2019)
Purpose: Search pre-computed sgRNAs, with genome browser and table views
Species Supported: *Drosophila*
URL: https://flyrnai-o2apps.hms.harvard.edu/crispr3/web/
Resource: SNP CRISPR (28)
Purpose: Find sgRNAs that specifically target single nucleotide polymorphic alleles
Species supported: *Drosophila*, human, mouse, rat, zebrafish
URL: https://www.flyrnai.org/tools/snp_crispr/web/

**Table 3.** Data-centric resources associated with the FlyRNAi database

Resource, purpose, species supported, URL
Resource: COMPLEAT (43)
Purpose: Protein complex enrichment analysis tool
Species supported: *Drosophila*, human, *S. cerevisiae*
URL: https://www.flyrnai.org/compleat/
Resource: SignedPPI (44)
Purpose: View PPI networks with edge signs annotated based on experimental data sets or predictions
Species supported: *Drosophila*
URL: https://www.flyrnai.org/SignedPPI/
Resource: DirectedPPI (45)
Purpose: View PPI networks with edge signs
Species supported: human
URL: https://www.flyrnai.org/DirectedPPI/
Resource: InsulinNet (46)
Purpose: Visualization of a comprehensive *Drosophila* InR/PI3K/Akt network generated in the Perrimon lab
Species supported: *Drosophila*
URL: https://fgrtools.hms.harvard.edu/InsulinNetwork/
Resource: *Drosophila* Gene Expression Tool (DGET) (29)
Purpose: Search and visualization of public bulk RNAseq data sets
Species supported: *Drosophila*
URL: https://www.flyrnai.org/tools/dget/web/
Resource: Molecular Interaction Search Tool (MIST) (32)
Purpose: Search and visualization of protein-protein interactions, interologs, and genetic interactions
Species supported: *C. elegans*, *Drosophila*, mouse, rat, *S. cerevisiae, S. pombe, X. laevis, X. tropicalis*, zebrafish
URL: https://fgrtools.hms.harvard.edu/MIST/
Resource: iProteinDB (36)
Purpose: Search and view of post-translational modifications such as phosphorylation sites
Species supported: multiple species in the *Drosophila* genus
URL: https://www.flyrnai.org/tools/iproteindb/web/
Resource: DRSC single-cell RNAseq (scRNAseq) data portal (30,31)
Purpose: Search and visualization of scRNAseq data sets generated by the Perrimon lab
Species supported: *Drosophila*
URL: https://www.flyrnai.org/scRNA/
Additional data sets for view and download, e.g. MitoMax mitochondrial proteomics data (47), *Drosophila* CRISPR knockout cell screen data (21,48)
URL: https://fgr.hms.harvard.edu/publications/publication-type/dataset-or-data-portal

### Gene-centric resources: from ortholog prediction to functional discovery

DIOPT integrates results from multiple ortholog prediction algorithms at a single, easy-to-use online interface (2). Since the launch of DIOPT in 2011, this online resource has been used extensively, with about 20,000 accesses per month. In addition, DIOPT-based ortholog mapping has been integrated into FlyBase (4), MARRVEL (5), and the Alliance of Genome Resources (6,7), and linked to from other resources such as gene report pages at PomBase (8). Since our 2017 update, we have expanded DIOPT to include *Arabidopsis thaliana* and results from three new ortholog prediction algorithms, OrthoFinder (9), OrthoInsepctor (10), and Hieranoid (11), bringing the total number of species supported to 10 and the total number of algorithms integrated at DIOPT to 17. Through user feedback, we became aware that there are ortholog pairs that have been experimentally demonstrated or for which other compelling evidence of an ortholog relationship exists but are not found in the imported mapping. For example, human LEPTIN (167 amino acids) can rescue a mutation in the *Drosophila* cytokine *unpaired 2*, suggesting a functional homology relationship between the two genes (12). In our experience, all user-suggested orthologous relationships involve fairly small proteins, particularly small open reading frames (ORFs). We suspect that this might reflect a limitation of current computational algorithms. To address gaps, we added a new page at DIOPT that makes it possible for users to submit orthologous relationships missing from database. The same page also makes it possible for users to submit comments about ortholog pairs that are included. New releases to DIOPT are launched about once per year. The current version (version 8) was launched in Sep 2019. In that version, we updated the gene annotations and integrated the most recent predictions from each source at that time. We expect to release version 9 by the end of 2020.

Identification of orthologs is just one step towards using ortholog information to develop new hypotheses regarding gene function. Information about each ortholog in a pair or group is typically found in a different model organism database (MOD), such as the Mouse Genome Information (MGI) database of mouse gene information (13) or the ZFIN database of zebrafish gene information (14). To help users quickly survey information about orthologous genes in all common genetic model organisms and humans, we implemented Gene2Fuction (15). Gene2Function helps users not only identify orthologs at different stringency levels based on DIOPT scores, but also summarizes information about genes across different species in a table format. The information presented in at Gene2Function includes evidence-based gene ontology annotations, publications, phenotypes, interactions, and expression in various tissues, information that is collected from each MOD using InterMine APIs (16,17).

To further help users gain information about a gene of interest, we also launched an advanced literature mining tool, BioLitMine (https://www.biorxiv.org/content/10.1101/2020.07.17.208249v1). When a user does a gene search at BioLitMine, the tool summarizes the medical subject heading (MeSH) index terms associated with the gene, allowing users to gain a big picture of what is reported in the published literature. BioLitMine can also be used to identify genes associated with a given MeSH term, identify genes frequently co-cited with an input gene, and build a co-citation network.

One important way that researchers engage in cross-species studies is by collaborating with experts in another system. To help facilitate identification of relevant experts, both Gene2Function and BioLitMine summarize the last-author information from all relevant publications for a given input gene. Furthermore, these resources display to users the number of papers associated with that gene and author, as well as the year of the most recent relevant paper and address listed in that publication. In addition, both Gene2Function and BioLitMine can help users build gene lists based on a topic. Specifically, Gene2Function can help users find genes related to a given human disease based on Online Mendelian Inheritance in Man (OMIM) annotations, and BioLitMine retrieves genes associated in the literature with a given MeSH term, including anatomy terms such as ‘stem cells,’ disease terms such as ‘breast cancer,’ and several additional categories.

### Reagent-centric resources: CRISPR as a new focus

**Figure 2.**
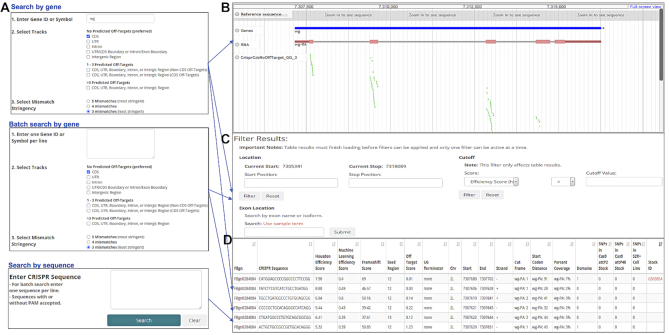
Features of the Find CRISPRs 3 online resource results page. (**A**) Search page with three search entry options: one gene, multiple genes or sgRNA sequences. (**B**) Visualization using the genome browser. (**C**) Filtering by genome location, exon or score. (**D**) Searchable, exportable and sortable table of all relevant sgRNA designs synchronized with the genome browser view.

CRISPR genome engineering technology is now widely used in research, including in *Drosophila* and other species. In 2013, we made available our first-generation CRISPR single guide RNA (sgRNA) design resource for *Drosophila*, Find CRISPRs (18), a searchable database of pre-computed sgRNA designs that includes a genome browser-based user interface. In 2015, we replaced the original tool with an improved version incorporating efficiency prediction scores based on in-house *Drosophila* data (18,19) with the same style of genome browser-based user interface (currently referred to as the ‘2017’ version, reflecting the last update). A limitation of these first two versions of the resource is that users can only query one gene at a time and have to click through all sgRNA designs to find an optimal design. More recently, we launched Find CRISPRs 3, the third version of the resource (currently referred to as the ‘2019’ version, reflecting the last update). This version combines the genome browser view with a table of all relevant designs. The results displayed in the table can easily be filtered or sorted. If a specific genome region such as one specific exon is selected by the user, the genome browser view will change, automatically zooming in on the selected exon and filtering table results. Moreover, in the third generation Find CRISPRs resource, we included an additional efficiency prediction score based on a machine learning approach applied to a set of genome-wide *Drosophila* cell CRISPR screen data (20,21). In this version, we also added protein domain annotations, as this information can be relevant in choosing an optimal target site for a given application, such as CRISPR knockout via non-homologous end joining (NHEJ) (22,23). We also sought to address the problem of CRISPR failure due to variants in the actual target genome as compared with the reference genome used to pre-compute the designs. To help address this for CRISPR knockout screens in *Drosophila* cells and specific *in vivo* studies, we incorporated SNP data from *Drosophila* S2R+ cultured cells, TRiP injection stocks (24), and CRIMiC injection stocks (25,26) into the latest version of resource. In addition, we annotated the reading frames at the cutting site for each relevant transcript to facilitate the design of CRISPR knock-in approaches (27). From the perspective of high-throughput screening projects, the most important feature added in the ‘2019’ version of Find CRISPRs was the ability to do a batch query to retrieve all CRISPR sgRNA designs for multiple genes or to retrieve information (e.g. efficiency scores) for multiple sgRNA sequences (Figure 2).

To help scientists design CRISPR sgRNAs targeting non-reference alleles, we also developed an alternative design tool, SNP-CRISPR (28). With this tool, users can specifically design sgRNAs that will target regions different from the reference genome with user-inputted information about the locations of genome variations in the specific genome they aim to target.

In recent years, the TRiP has changed the transgenic fly stock production of RNAi to sgRNA for knockout or activation purposes (24). To support this pipeline, we implemented a new LIMs system to track the production process of making transgenic fly stocks from gene nomination, sgRNA design and primer ordering to construct making and homozygosing. There are two portals with different access levels to the LIMs system. The public portal allows the community to nominate genes, check the progress of nomination, and search for available fly stocks, while the internal portal is password protected and allows the production team to find or update information at different production stages. Currently the pipeline has processed 7734 nominations, of which 1748 were from the community, and has obtained stocks for 4380 requests.

We originally developed the RNAi Stock Validation and Phenotype (RSVP) tool to track phenotype and qPCR validation data for RNAi transgenic stocks. Our goals in collecting this information were to help researchers to identify optimal in vivo RNAi stock(s) when multiple reagents are available, to identify stocks that do not perform well and thus could be culled from stock collections, and to identify genes for which effective fly stock reagents are lacking. This type of resource also applies to fly stocks developed for tissue-specific application of CRISPR approaches, including stocks developed for knockout and activation. Recognizing that researchers are likely to want to search for RNAi and CRISPR fly stock reagents together rather than separately, we decided to expand RSVP to include information about CRISPR fly stocks rather than support this in a separate resource. To accommodate CRISPR fly stock information, we modified the resource from support of an ‘enhancer-Gal4 + UAS-RNAi reagent’ structure to include a third component, ‘enhancer-Gal4 + UAS-sgRNA reagent (or U6-sgRNA reagent) + UAS-Cas9 or a UAS-dCas9::Activator,’ in order to accurately reflect the design of CRISPR-based *in vivo* studies. To do this, we upgraded the database design and user interface and renamed the resource ‘RSVP Plus’ (24).

### Data-centric resources: new data types and enhanced integration of different data types

With the development of new technologies and new public data resources, more and more data types become publicly available. In response, the DRSC informatics group has been actively adding new data mining tools for a variety of data types. For example, we developed DGET for data mining of bulk RNA-seq data (29) and recently added a single-cell RNAseq (scRNAseq) portal that allow scientists to mine data sets generated by the Perrimon lab (30,31). At this new data portal, users can view cell-level expression on t-distributed stochastic neighbor embedding (tSNE) or uniform manifold approximation and projection (UMAP) visualizations of all conditions or a specific condition. Users can also visualize cluster-based results with a violin plot, dot-plot, or heatmap for a given set of genes of interest. A batch download option is also available for cluster-based results as well as marker gene information with fold changes and *P* values.

The availability of protein–protein interaction or genetic interaction data has increased dramatically in recent years and there are many public resources that make such data available. To take advantage of this, in 2018 we launched MIST (Molecular Interaction Search Tool) (32), which integrates interaction data from public repositories such as BioGrid (33) and IntAct (34) as well as from literature curation efforts such as by FlyBase (4) and WormBase (35). What makes MIST unique compared to other resources is both the comprehensiveness of the data and annotation at MIST of interologs, i.e. predicted interactions based on ortholog mapping. To provide interologs, MIST maps interaction data among model organisms using DIOPT. As a result, users can build networks at MIST that are based on data from an entry species and data from other species.

In 2019, DRSC also launched iProteinDB, which allows users to mine information about post-translational modifications (PTMs) generated by the Perrimon lab and collaborators for *Drosophila*, as well as by other groups (36). The iProteinDB resource includes and compares PTM data (specifically, phosphorylation data) for six closely related species in the *Drosophila* genus. In addition, users can align a *Drosophila* protein of interest with its orthologs in other major model organisms (human, mouse, rat, frog, zebrafish, worm) and compare *Drosophila* PTM data with PTM data available for orthologs.

### Summary and future directions

Since our 2017 update, the DRSC informatics group has focused on three main areas. First, we developed gene-centric tools that take advantage of our ability to use DIOPT to map orthologs, allowing us to integrate annotation information for orthologs of multiple species in unified interfaces, resulting in Gene2Function and BioLitMine. Second, we shifted our focus with regards to reagent design and phenotype tracking from RNAi to CRISPR, for example by adding CRISPR-Cas9 data to what we now call RSVP Plus and improving our Find CRISPRs resource. Third, we expanded our resources to include more data types, such as expression and protein information, allowing us to provide support for data mining based on bulk RNAseq and scRNA-seq data sets, as well as for protein-focused data, including interaction data (MIST) and protein post-translational modification data (iProteinDB).

We specifically enhanced data integration in two ways. First, we integrated additional data types, for example, integration of tissue-specific expression data with interaction data at MIST, allowing users to identify tissue-specific interaction partners and build tissue-specific networks. Second, we now facilitate comparison and integration of equivalent data types across species, using DIOPT for ortholog mapping. For example, users can view both interaction data from a species of interest and predicted interactions based on other species (interologs) on a single, color-coded network at MIST. Another example is provided by iProteinDB, in which amino acids that have been identified as post-translationally modified in different species are color-coded on a multiple-sequence alignment, such that PTM data for all orthologs are displayed together and thus, can be compared.

Our future directions will focus on development of data-centric tools that improve our current resources and expand coverage for even more data types, such as ChIP-seq, ATAC-seq and Hi-C data. With more and more data becoming available, there is need for new data integration and analysis tools. We anticipate that data mining and data analysis tools tailored for model organism research will be a major focus for our group in the future. For example, in the area of data mining, we anticipate developing ways to make use of the wealth of scRNAseq data sets that are being generated for *Drosophila* and other species. We are also looking for areas in which we can build upon our experience and infrastructure to provide gene-, data-, and reagent-centric support for additional species, such as mosquito vectors of infectious diseases.

## DATA AVAILABILITY

All the online informatics tools/resources can be found at https://fgr.hms.harvard.edu/tools.
